# Insecticide resistance status of *Aedes aegypti* and *Aedes albopictus* mosquitoes in southern Benin, West Africa

**DOI:** 10.1186/s41182-023-00514-y

**Published:** 2023-04-21

**Authors:** Alphonse Keller Konkon, Germain Gil Padonou, Razaki Osse, Albert Sourou Salako, David Mahouton Zoungbédji, Haziz Sina, Arthur Sovi, Filemon Tokponnon, Rock Aïkpon, Herbert Noukpo, Lamine Baba-Moussa, Martin Codjo Akogbéto

**Affiliations:** 1 Centre de Recherche Entomologique de Cotonou (CREC), 06 BP 2604, Cotonou, Benin; 2 Faculté des Sciences et Techniques de l’Université d’Abomey-Calavi, Abomey-Calavi, Benin; 3 École de Gestion et d’exploitation des Systèmes d’élevage de l’Université Nationale d’Agriculture de Porto-Novo, Porto-Novo, Benin; 4 Department of Biochemistry and Cellular Biology, Laboratory of Biology and Molecular Typing in Microbiology, University of Abomey-Calavi, Abomey-Calavi, Benin; 5 Faculty of Agronomy, University of Parakou, Parakou, Benin; 6 Faculty of Infectious and Tropical Diseases, The London School of Hygiene and Tropical Medicine, London, UK; 7 Ministère de la Santé, 08 BP 882, Cotonou, Benin; 8 Université Nationale des Sciences, Technologies, Ingénierie Et Mathématiques (UNSTIM), Abomey, Benin

**Keywords:** *Aedes aegypti*, *Aedes albopictus*, Insecticide resistance diagnostics, Benin

## Abstract

**Background:**

The emergence of insecticide resistance in *Aedes* mosquitoes could undermine efforts to control arboviruses. The present study aims to assess in some communes of Southern Benin, the susceptibility level of *Aedes aegypti* (Linnaeus, 1762) and *Aedes albopic*tus (Skuse, 1894) to insecticides commonly used in public health, as well as mechanisms involved.

**Methods:**

Females *Ae. albopictus* and *Ae. aegypti* collected in Ifangni, Porto-Novo, Avrankou, Adjarra and Kétou from June 2021 to October 2022, were exposed to: deltamethrin 0.05%, permethrin 0.75%, alpha-cypermethrin 0.05%, pirimiphos methyl 0.25% and bendiocarb 0.1%, following the standard WHO susceptibility tube test protocol. In some sites, pre-exposure to the synergist PBO was used to verify if pyrethroid resistance of populations of *Aedes* was mediated by oxidases.

**Results:**

Full susceptibility to deltamethrin and permethrin was observed in all tested populations of *Ae. albopictus*. However, with alphacypermethrin, a suspected resistance was observed in Adjarra (94.67%), Ifangni (93%) and Porto-Novo (94%), and a resistance in Avrankou (83%). The PBO-alphacypermethrin tests performed, led to a full susceptibility (100%) in all four sites, which confirms the full involvement of oxidases in resistance of all tested populations of *Ae. albopictus* to alphacypermethrin. At the opposite, *Aedes aegypti* was either resistant or suspected of being resistant to all tested pyrethroids in all four sites, except in Ifangni where a full susceptibility to alphacypermethrin was observed. The full susceptibility of *Ae. aegypti* to bendiocarb and pirimiphos-methyl in all communes suggests that these two insecticides can be good candidates for an effective control of pyrethroid-resistant *Aedes* vector populations. Use of permethrin and deltamethrin could also be considered for controlling populations of *Ae. albopictus*.

**Conclusion:**

Results of the present study will help guide strategy to implement for an effective control of *Aedes* vector populations in Benin.

## Background

*Aedes aegypti* is the main indigenous vector of arboviruses in Africa [1]. In Benin, it remains abundant in both the south and the north parts of the country [2, 3]. Recent studies have reported the presence of *Aedes albopictus*, a vector native to Asia, in Southern Benin [4, 5]. Indeed this mosquito species is known to be invasive, and was involved in several cases of arbovirus epidemics [6, 7]. Dengue which was considered more prevalent in Asia and Latin America, has now spread to several West African countries [8], where all four serotypes of the virus are actively circulating [9]. This worrying situation deserves more attention, as severe forms of the disease are often found in areas where more than two serotypes of the virus coexist [10, 11].

Several cases of dengue fever have been diagnosed in European tourists returning from Benin in recent years [12–14]. Indeed, those travelers have tested positive for DENV-1, a virus serotype native to Asia [14]. Moreover, DENV-3 was recently detected in a sample of *Aedes aegypti* from Porto-Novo, the political capital of Benin [15].

Several factors make Benin a country likely to experience an arbovirus epidemic. These factors include: the high demographic growth, the development of trade of second hand cars and tires, the development of tourism, the presence of two major vectors of arboviruses, the growing urbanization of the country, and its proximity with Nigeria, an endemic country with which it shares a 773 km border. Except for yellow fever for which an effective vaccine is available, the other arboviruses have no curative or preventive treatment [16]. As a result, vector control appears to be the only way to control the disease transmission [17]. This control could be through the use of chemicals for killing immature stages or adult mosquitoes, the destruction of vector breeding sites resulting in the reduction of their density, and the sensitization of the population on good practices for water conservation and sanitation. Even with a strong community engagement, it is quite difficult to identify and destroy all breeding sites in the environment. Therefore, chemical control through the implementation of spatial spraying, and use of long lasting insecticidal nets (LLINs) may be the most effective way to control dengue vectors. Several studies have recently reported resistance of *Aedes aegypti* to pyrethroids and carbamates in Africa and Asia [18–20]. *Ae. aegypti* is well adapted to urban habitats and, therefore, is generally more susceptible to insecticide exposure and resistance than *Ae. Albopictus*[21].In West Africa, most of the available data on resistance of *Aedes* mosquitoes to insecticides, are for *Ae. aegypti* only, despite the reported introduction of *Ae. albopictus* in Nigeria since 1971. Recently, 238 and 380 confirmed cases of dengue fever have been recorded in Senegal and Côte d'Ivoire, respectively [22, 23]. To plan an effective and sustainable control strategy against arbovirus vectors in Benin, there is an urgent need to determine the susceptibility level of *Ae. albopictus* to insecticides commonly used in public health, and update data on the resistance status of *Ae. aegypti*. The present study aims to assess the resistance profile of these two main vectors of arboviruses, in Benin.

## Methods

### Study area

**Fig. 1:**
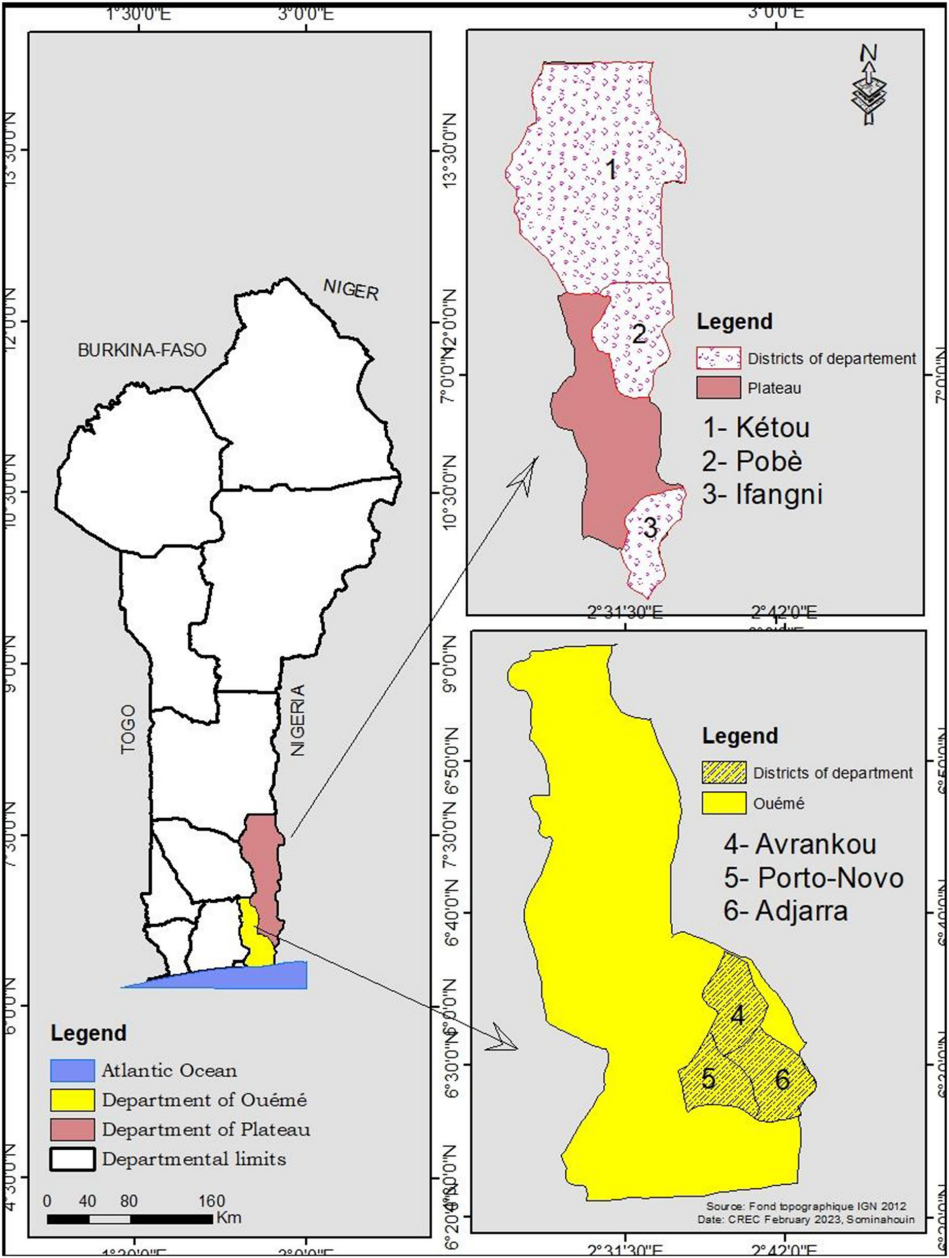
Map showing the study area

The study was carried out in the urban and peri-urban areas of 6 communes belonging to two departments in Southern Benin. These are the communes of Avrankou, Adjara, and Porto-Novo in the department of Ouémé and the communes of Kétou, Ifangni and Pobè in the department of Plateau (Fig. 1). These communes that mostly neighbor Nigeria, were surveyed because of the presence recently reported of *Ae. albopictus* and *Ae. aegypti* [6]. The whole study area is characterized by a subequatorial climate with two rainy seasons (March to July, and September to November) and two dry seasons (November to February and July to August) with rainfall ranging from 1200 to 1500 mm/year. There are three groups of ecosystems, namely, coastal plain ecosystems, bar land plateau ecosystems and Lama depression clay ecosystems. Long-lasting insecticidal nets (LLINs) distributed every three years throughout the country, and repellents are the main vector control tools used in the study area.

### Mosquito collection

Three sampling techniques were used to collect immature stages and adults of *Aedes spp.* from July 2021 to October 2022. Irrespective of the collection method, the morphological identification of adult mosquitoes was performed using the taxonomic keys of Edwards [24] and Huang & Rueda [25].

### Larvae collection

Immature stages of *Aedes spp.* were sampled from various breeding sites (abandoned jars, tires, cans, and other containers) using the dipping technique. They were filtered and stored in labeled jars, and then transported to the insectary of the Centre de Recherche Entomologique de Cotonou (CREC) for rearing until adulthood.

### Ovitrapping method

The ovitraps were used as part of the present study. They were made of polyethylene bottles which were filled with 50 cl of water. A hardboard plate (5 cm by 20 cm) immersed into the water, served as support for the eggs laid. A total of 12 ovitraps were set per site. These traps were hung on a tree/wall at a height of 1.5 m from the ground, using a nail and a metal string, and left for one week in the domestic or peri-domestic environment. To avoid egg-hatching, the inspection of traps occurred on a daily basis. They were removed after 7 days, and the eggs laid on the hardboard plates were brought back to the insectary and put in water. After the hatching, the larvae were reared until adult stage.

### Human landing catches

This method was used to collect adults of *Aedes* spp. during the day. In all study communes, mosquito collections were conducted both indoors and outdoors, with a first group of collectors that worked from 7:00 am to 1:00 pm and which was replaced by a second group from 1:00 pm to 6:00 pm. At each collection point, a volunteer with bare-legged and barefoot serving as bait, collected mosquitoes using hemolysis tubes and a flashlight. The specimens of *Aedes spp*. that were collected, were released in cages and transported to the insectary.

### Biological materials

Females *Ae. aegypti* field-collected as larvae, were used for the WHO susceptibility tube testing.

For *Ae. albopictus,* mosquitoes from the three collection methods were reared at the insectary, in order to have a sufficient number of individuals of F1 generation. Only the females mosquito of this generation, were used for the WHO susceptibility tube testing.

### Insecticide susceptibility tests

WHO susceptibility tube tests were performed according to the WHO protocol [26], using non-blood-fed females *Ae. aegypti* (F0) and *Ae. albopictus* (F1), aged 2–5 days. These mosquitoes were exposed to the following products:

-deltamethrin (0.05%), permethrin (0.75%), and alphacypermethrin (0.05%);
-alphacypermethrin (0.05%) + PBO (4%).

Batches of 20–25 mosquitoes were introduced into each tube carpeted with an insecticide-treated paper for a 1-h exposure. The number of mosquitoes knocked down by the insecticide at different time intervals (5, 10, 15, 20, 30, 45, 60 min) was recorded. A batch of 20–25 mosquitoes exposed to an insecticide-free paper was used as a control.

The PBO synergist is an inhibitor of oxidases [27]. It helped assessing the involvement of oxidases in the pyrethroid resistance observed in the populations of *Aedes*. The PBO-pyrethroid, and pyrethroid-only tests were performed simultaneously on the same mosquito populations. After 60 min of exposure, mosquitoes were transferred into observation tubes and kept at 25 °C and 80% humidity, with free access to a 10% sweetened juice. Mortality after 24 h was determined according to the WHO protocol.

### Biochemical analyses

Thirty females *Ae. aegypti* from each district, aged 2–5 days, and non-previously exposed to any insecticide, were used for biochemical analyses. These were performed to compare the expression level of detoxification enzymes (mixed function oxidases, non-specific esterases and glutathione S-transferases) of different populations of *Aedes aegypti* to the reference susceptible strain (Rockefeller), following the protocol described by Hemingway et al. [28].

### Data analysis

Mortality rates from the susceptibility tube tests were interpreted according to the WHO protocol [26]. When a mosquito population had a mortality rate between 98 and 100%, it was considered susceptible. When mortality was between 90 and 97%, the population was suspected of resistance. Below a mortality rate of 90%, the population was said resistant. The 24-h mortality rates and the 60-min knockdown rates were compared using the Chi-square test for comparison of proportions. A linear regression with analysis of variance was used to assess the variation in enzyme activity for each mosquito population. The Mann–Whitney *U* test was used to compare the enzyme activity between the field mosquito populations and the susceptible laboratory strain (Rockefeller). Statistical analyses were performed using R 3.3.2 software [29].

## Results

### Mortality and knockdown rates

In total, 3517 females *Aedes* (1787 *Ae. aegypti,* and 1730 *Ae. albopictus*), were bioassayed.

Table 1 shows the pyrethroid-induced mortalities in the different *Aedes* mosquito populations. After 60 min of exposure to the different insecticides, the knockdown rates observed in *Ae. albopictus* was very high in all communes, ranging between 94 and 100% (Table 1). A 100% mortality rate was observed in the different populations of *Ae. albopictus* exposed to deltamethrin and permethrin, which indicates a full susceptibility of these populations to the two tested pyrethroids (Table 1). Similarly, the population of *Ae. albopictus* from Kétou showed full susceptibility to alphacypermethrin (100%). However, with the same product, a suspected resistance was observed in Adjarra (94.67% [86.19–98.27]), Ifangni (94.38% [86.78–97.91]) and Porto-Novo (95.7% [88.73–98.61]), and a resistance in Avrankou (83% [73.89–89.50]) (Table 1).

**Table 1:** Mortality observed 24 h after exposure of *Aedes albopictus* to permethrin, deltamethrin and alpha-cypermethrin

Insecticides	Districts	Nb of *Aedes albopictus* tested	Mean knockdown after 60 min (%) (95% CI)	Mean mortality (%) after 24 h (95% CI)	Resistance status
Alpha-cypermethrin (0.05%)	Avrankou	100	100 [95.38–100]	83 [73.89–89.50]	R
	Adjarra	75	100 [93.92–100]	94.67 [86.19–98.27]	SR
	Porto-Novo	93	95.7 [88.73–98.61]	95.7 [88.73–98.61]	SR
	Kétou	89	100 [94.84–100]	100 [94.84–100]	S
	Ifangni	89	94.38 [86.78–97.91]	94.38 [86.78–97.91]	SR
Deltamethrin (0.05%)	Avrankou	92	100 [95.00–100]	100 [95.00–100]	S
	Adjarra	100	100 [95.38–100]	100 [95.38–100]	S
	Porto-Novo	93	100 [95.05–100]	100 [95.05–100]	S
	Kétou	90	100 [94.89–100]	100 [94.89–100]	S
	Ifangni	90	100 [94.89–100]	100 [94.89–100]	S
Permethrin (0.75%)	Avrankou	93	100 [95.05–100]	100 [95.05–100]	S
	Adjarra	85	100 [94.61–100]	100 [94.61–100]	S
	Porto-Novo	94	98.93 [93.37–99.94]	100 [95.10–100]	S
	Kétou	95	100 [95.15–100]	100 [95.15–100]	S
	Ifangni	95	100 [95.15–100]	100 [95.15–100]	S

*Nb* number, *R* resistance, *SR* suspected resistance, *S* susceptibility

In the populations of *Ae. aegypti*, a different trend was observed, with much more variable 60-min knockdown rates. Indeed, irrespective of the pyrethroid insecticide, these rates ranged from 63.51% [51.45–74.16] in Porto-Novo to 100% [95.29–100] in Ifangni (Table 2). 24-h post-exposure, populations of *Ae. aegypti* from Avrankou (91% [83.16–95.54]), Adjarra (93.75% [85.38–97.67]), Porto-Novo (93.51% [84.89–97.58]) and Kétou (95.65% [88. 61–98.59]) showed a suspected resistance to alpha-cypermethrin (0.05%), while the one of Ifangni displayed a full susceptibility to the same insecticide (Table 2). With deltamethrin (0.05%), a suspected resistance was observed in Avrankou (95% [88. 17–98.14]), Adjarra (95.29% [87.73–98.48]), Ifangni (90.91% [83.00–95.49]), and Kétou (93.87% [86.62–97.48]), while there was a resistance to the same product in Porto-Novo (63.51% [51.45–74.16]). Furthermore, resistance to permethrin was observed in all tested populations of *Ae aegypti,* except for the Ifangni strain that displayed a suspected resistance (97% [90.84–99.22]) (Table 2).

**Table 2:** Mortality observed 24 h after exposure of *Aedes aegypti* to permethrin, deltamethrin and alpha-cypermethrin

Insecticides	Districts	Nb of *Aedes aegypti* tested	Mean knockdown after 60 min (95% CI)	Mean mortality after 24 h (95% CI)	Resistance status
Alpha-cypermethrin (0.5%)	Avrankou	100	91.00 [83.16–95.54]	91.00 [83.16–95.54]	SR
	Adjarra	80	93.75 [85.38–97.67]	93.75 [85.38–97.67]	SR
	Porto-Novo	77	93.51 [84.89–97.58]	93.51 [84.89–97.58]	SR
	Kétou	92	95.65 [88.61–98.59]	95.65 [88.61–98.59]	SR
	Ifangni	98	100.00 [95.29–100]	100.00 [95.29–100]	SS
Deltamethrin (0.05%)	Avrankou	100	95.00 [88.17–98.14]	95.00 [88.17–98.14]	SR
	Adjarra	85	95.29 [87.73–98.48]	95.29 [87.73–98.48]	SR
	Porto-Novo	74	63.51 [51.45–74.16]	63.51 [51.45–74.16]	R
	Kétou	98	93.87 [86.62–97.48]	93.87 [86.62–97.48]	SR
	Ifangni	99	90.91 [83.00–95.49]	90.91 [83.00–95.49]	SR
Permethrin (0.75%)	Avrankou	99	89.89 [81.79–94.78]	89.89 [81.79–94.78]	R
	Adjarra	90	84.44 [74.93–90.93]	84.44 [94.89–100]	R
	Porto-Novo	84	77.38 [66.70–85.50]	77.38 [66.70–85.50]	R
	Kétou	95	84.21 [74.97–90.60]	84.21 [74.97–90.60]	R
	Ifangni	100	97 [90.84–99.22]	97 [90.84–99.22]	SR

*Nb* number, *R* resistance, *SR* suspected resistance, *S* susceptibility

**Fig. 2:**
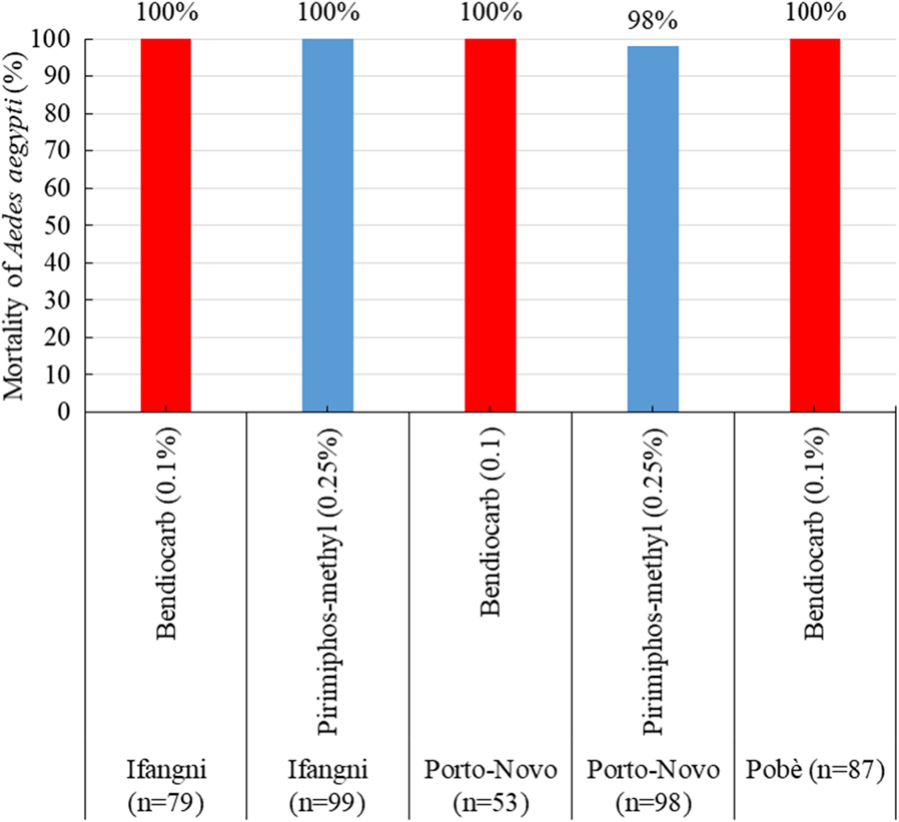
Mortalities observed 24 h after exposure of *Ae. aegypti* to bendiocarb and pirimiphos methyl

Figure 2 shows the mortality induced by bendiocarb and pirimiphos methyl. Overall, all tested populations of *Ae. aegypti* displayed a full susceptibility (mortality rates ≥ 98%) with these two products.

**Fig. 3:**
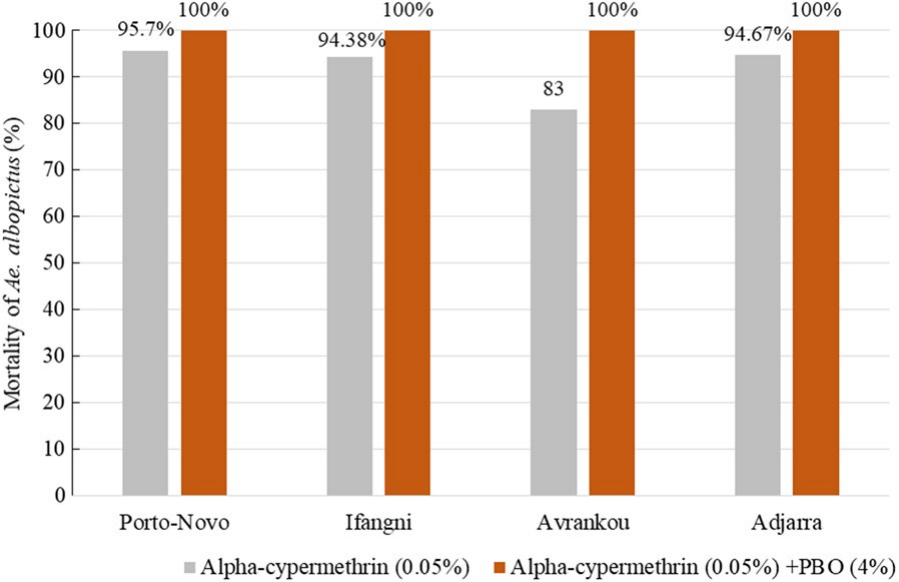
Mortality rates of *Aedes albopictus* with alpha-cypermethrin alone and alpha-cypermethrin + PBO

The PBO-alphacypermethrin tests performed with the four field strains of *Ae. albopictus* led to a mortality rate = 100% and higher than the one for the insecticide alone, indicating a full involvement of oxidases and esterases in alphacypermethrin resistance (Fig. 3).

### Expression of oxidases, esterases and GSTs in *Aedes aegypti*

**Fig. 4:**
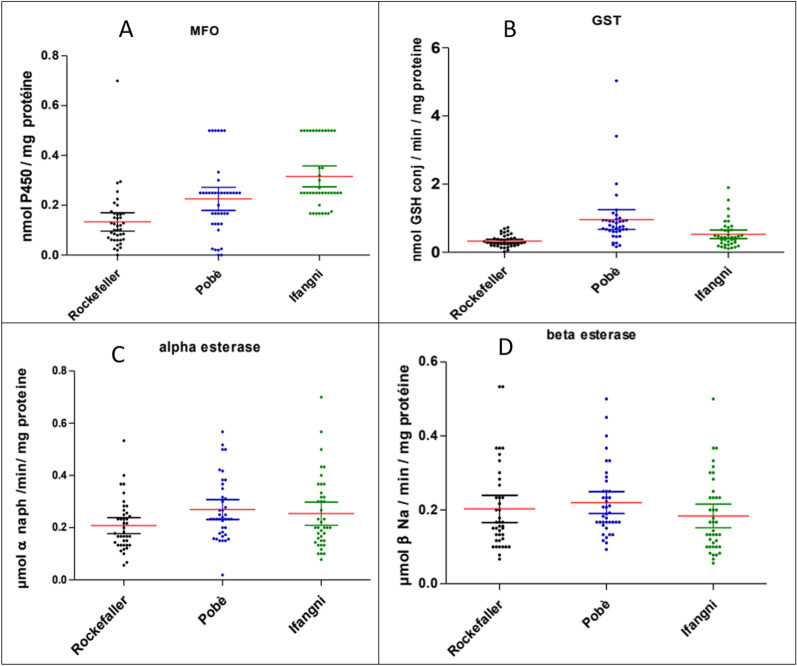
Activities of glutathione-S-transferase (**A**), mono-oxygenase (**B**), β-esterases (**C**), and α-esterases (**D**) in *Ae. aegypti* collected at Pobè and Ifangni

Figure 4 shows the mean levels of enzyme activities in the field populations of *Ae. aegypti,* and in the reference susceptible strain (Rockefeller).

The results show an overexpression of GSTs in the population of *Ae. aegypti* from Pobè (*P* < 0.0001) compared to the Rockefeller susceptible strain (Fig. 4A). A significantly elevated oxidase activity was observed in the populations of *Ae. aegypti* from Pobè and Ifangni, compared with that of the Rockefeller susceptible strain (*P* > 0.001) (Fig. 4B). In contrast, no overexpression of the α and β esterase activity was seen in all the tested populations compared with the Rockefeller susceptible strain (Fig. 4C, D).

## Discussion

Studies on insecticide resistance profile have long been performed in Benin in *Anopheles* vectors of malaria, but very little is done on *Aedes* responsible for arboviruses, even though the country is close to countries Nigeria, Ghana, Cote d’Ivoire, Burkina-Faso that in recent years have become dengue endemic. Recently, *Ae. albopictus*, a highly invasive Asian mosquito, has entered Benin and lives in sympatry with *Aedes aegypti* in urban and peri-urban areas [4]. Both species are described as the main vectors of arboviruses [3, 4]. The present study provides knowledge on their insecticide resistance profile, which will help to better orient the vector control strategy to implement in case of a dengue epidemic.

Findings of the present study showed full phenotypic susceptibility of the different populations of *Ae. albopictus* tested to permethrin and deltamethrin, despite the widespread resistance observed of *Anopheles* vectors and *Ae. aegypti* to these two pyrethroids in Benin [4, 30]. This could be because permethrin and deltamethrin are among the type II pyrethroids with high toxicity. However, with alpha-cypermethrin, *Ae. albopictus* was resistant in Avrankou, and suspected of resistance in Adjarra, Porto-Novo and Ifangni. This shows the beginning of the emergence of resistance of *Ae. albopictus* to alphacypermethrin, a type I pyrethroid with a relatively low toxicity compared to type IIs. Previous studies had already revealed a relative susceptibility of this mosquito species to pyrethroids [21]. This onset of resistance of *Ae. albopictus* to pyrethroids was reported by Ngo et al. [31] and Yougang et al. [32] in the cities of Douala and Yaoundé in Cameroon, respectively. According to these authors, this rapid expansion of resistance in *Ae. albopictus* could result from domestic or organic pollutants, as this species is widely distributed in water tanks, spare tires and discarded containers, which are widely distributed in cultivated agricultural sites [33]. It is also possible that the susceptibility of this species has been affected by the increased use of repellent insecticides in peri-urban settings. However, this resistance of *Ae. albopictus* to alpha-cypermethrin was reversed by the synergist PBO in all communes, confirming the involvement of oxidases in pyrethroid resistance in the localities studied [34]. The deployment of next-generation LLINs combining the PBO synergist with a pyrethroid insecticide could be considered an alternative option for an effective control of populations of Ae albopictus. At the opposite, *Ae. aegypti*was found to be much more resistant or suspected of being resistant to pyrethroids. Similar observations were previously made in Cameroon [32]. This supports the hypothesis of an occurrence of a strong insecticide resistance selection in *Ae. aegypti* relative to *Aedes albopictus*. It is likely that these species exhibit different biting and resting behaviors, which could explain variable insecticide exposure. Indeed, *Ae. aegypti* has been frequently collected indoors in urban environments, while *Ae. albopictus* is much more sampled outdoors in a peri-urban environment. This particular behavior of *Ae. aegypti* could expose it more to indoor insecticide-based interventions such as insecticide sprays, aerosols, or LLINs to prevent nuisance in urban settings [35–38]. This high resistance of *Ae. aegypti* compared to *Ae. albopictus* has been reported in different epidemiological settings in Central and West African countries [39–41] such as Benin [3]. Reduced susceptibility of *Aedes* mosquitoes to pyrethroids is widespread and has been reported in several countries [17]. Our results corroborate previous studies from Thailand that reported high resistance to deltamethrin and permethrin in *Ae. aegypti* [42, 43]. Our biochemical data revealed the overexpression of MFOs and GSTs in populations of *Ae. aegypti* from Ifangin and Pobè. Oxidases are involved in the detoxification of pyrethroids in mosquitoes [44, 45]. The enormous quantities of insecticides of the same class used in agriculture could be at the origin of this overproduction of oxidases. The glutathione-S-transferase activity observed in our vector populations confirms the strong resistance to DDT observed by Yadouleton et al. [2] in *Ae. aegypti* in Benin. High GST expression may be due to overexpression of the GST2 gene [45]. In contrast, the low esterase activity observed would certainly be because the vectors from Ifangni and Pobè have not been or are weakly exposed to carbamates and organophosphates. However, a full susceptibility of populations of *Ae. aegypti* to bendiocarb and pyrimiphos-methyl was observed in all the surveyed communes. This can be explained by the low esterase activity observed in Ifangni and Pobè, where the use of pyrimiphos methyl and carbamates is low. It would be also interesting to characterize the *Kdr* and *Ace-1R* resistance mechanisms in populations of Aedes vectors, as they are commonly found in Anopheles mosquitoes. Carbamates and organophosphates could also be considered a good alternative for the control of arbovirus vectors in Benin in case of epidemics. Though our findings suggest that permethrin and deltamethrin could also be used, there is a growing concern that *Ae. albopictus* develop resistance to these pyrethroid insecticides. *Ae. aegypti* may develop other resistance mechanisms, such as behavioral or physiological one, to circumvent the modes of action of these insecticides. It would be interesting to investigate all the resistance mechanisms in *Ae. aegypti* to establish its full resistance profile to make good vector control decisions.

## Conclusion

The present study showed full susceptibility of *Ae. albopictus* to deltamethrin and permethrin but widespread resistance to pyrethroids in *Ae. aegypti* in the study area. This suggests that these insecticides (deltamethrin and permethrin) can be used in arboviruses control programs in sites where *Ae. albopictus* is reported as the main vector. Given pyrethroids resistance observed in *Ae. aegypti,* and the emergence alpha-cypermethrin resistance in *Ae. albopictus*, rational use of insecticides, especially pyrethroids, should be encouraged to help reduce insecticide pressure. The synergist PBO increased the mortality of *Ae. albopictus* to alpha-cypermethrin, showing the involvement of oxidases. However, full susceptibility of *Ae. aegypti* to bendiocarb and pirimiphos methyl was observed in all study communes.

Given the increasing number of dengue cases in neighboring countries, strategies to improve vector control and prevent the spread of the diseases in Benin are urgently needed.

## Data Availability

The datasets that were analyzed in this study are available from the corresponding author and the lead author.

## Abbreviations

CREC: Centre de recherche entomologique de cotonou
WHO: World Health Organization
LLINs: Long-lasting insecticidal nets
GST: Glutathione-S-transferase
DDT: Dichlorodiphenylchloroethane
MFO: Mixed function oxygenases
PBO: Piperonyl butoxide
HLC: Human landing catches
